# MEETINGS CALENDAR - 2017

**Published:** 2017

## October

### ► 1 - 5 to 7 - Heart Valve Summit: Medical, Surgical and Interventional Decision Making

Chicago, United States

Informations: AATS

Phone: 978-252-2200

Fax: 978-522-8469

E-mail: meetings@aats.org

Site: aats.org/valve

### ► 5 to 7 - 14th Annual Multidisciplinary Cardiovascular and Thoracic Critical Care Conference

Washington, United States

Informations: Michelle Taylor

Phone: 312-202-5800

Fax: 312-202-5801

E-mail: education@sts.org

Site: sts.org/criticalcare

### ► 5 and 6 - Cardiovascular Disease Management: A Case- Based Approach, 5th Annual Symposium

Phoenix, United States

Informations: Promedica International CME

Phone: 760-720-2263

Fax: 760-720-6263

Site: promedicacme.com

### ► 6 and 7 - 4th Annual Advances in Thoracic Surgical Oncology

New York, United States

Informations: Office of Continuing Medical Education

Phone: 646-227-2025

E-mail: cme@mskcc.org

Site: mskcc.org/ThoracicSurgeryCourse

### ► 7 to 11 - 31st EACTS Annual Meeting

Vienna, Austria

Informations: EACTS

E-mail: info@eacts.co.uk

Site: eacts.org/educational-events/eacts-annual-meeting

### ► 13 - Pulmonary Hypertension

Toronto, Canada

Informations: Jennifer O'Hare

E-mail: info-MED1778@cpdtoronto.ca

Site: cpd.utoronto.ca/pulmonaryhypertension

### ► 16 to 18 - 20th European Cardiology Conference

Budapest, Hungary

Informations: David Mathews

Phone: 7025085200

E-mail: eurocardiology@cardiologyconference.org

Site: cardiology.conferenceseries.com/europe

### ► 18 to 21 - ECTSS 55th Annual Meeting

Amelia Island, United States

Informations: Eastern Cardiothoracic Surgical Society

Phone: 01296 733 823

Fax: 01296 733 823

E-mail: meeting@ectss.org

Site: ectss.org

### ► 20 and 21 - 6th Global Experts Meeting on Cardiology and Cardiovascular Pharmacology

Osaka, Japan

Informations: Vaishnavi N

Phone: 7025085200

E-mail: cardiacpharmacology@conferenceseries.net

Site: cardiac.pharmaceuticalconferences.com

### ► 20 and 21 - International VATS Symposium 2017

London, United Kingdom

Informations: Lorraine Richardson, Mr. Marco Scarci

Phone: 01296 733 823

Fax: 07711 132 946

E-mail: lorrainerichardson1@btinternet.com marco.scarci@mac.com

Site: www.internationalvats.com

### ► 21 and 22 - Endovascular Skills Course

Hamburg, Germany

Informations: EACTS

E-mail: info@eacts.co.uk

Site: eacts.org/educational-events/programme

### ► 23 to 27 - Fundamentals in Cardiac Surgery: Part III

Windsor, United Kingdom

Informations: EACTS

E-mail: info@eacts.co.uk

Site: eacts.org/educational-events/programme

